# Successful treatment of infectious endocarditis-associated glomerulonephritis during active hepatitis C infection: a case report

**DOI:** 10.1186/s12882-022-02985-3

**Published:** 2022-12-07

**Authors:** Anna Zito, Antonio De Pascalis, Vincenzo Montinaro, Paolo Ria, Maria Caterina Carbonara, Emiliana Ferramosca, Marcello Napoli

**Affiliations:** 1 Department of Nephrology, Vito Fazzi Hospital, Lecce, Italy; 2 Department of Nephrology, Miulli General Hospital, Acquaviva delle Fonti, Bari, Italy

**Keywords:** Hepatitis C infection, Endocarditis, Cryoglobulinemia, Membrano-proliferative glomerulonephritis, Renal biopsy, Case report

## Abstract

**Background:**

Hepatitis C virus (HCV) may play a pathogenic role in several forms of immune complex glomerulonephritis (GN). We present a patient whose initial clinical presentation instilled suspicion of HCV-related renal involvement. Yet, histopathologic data oriented towards a different diagnosis.

**Case presentation:**

A 68-year old man presented with kidney dysfunction, cryoglobulins, low C4 level, high HCV—RNA and cutaneous vasculitis. The first hypothesis was a hepatitis C-related cryoglobulinemic glomerulonephritis. Renal biopsy revealed endocapillary and mesangial cells hypercellularity with complement C3 and IgM deposits. The echocardiography showed an infectious endocarditis (IE) on aortic valve. Appropriate antibiotic therapy and a prosthetic valve replacement were performed, obtaining recovery of renal function.

**Conclusion:**

HCV infection may be linked to multiple renal manifestations, often immune-complex GN such as cryoglobulinemic membrano-proliferative GN. Renal disease due to IE is usually associated to focal, segmental or diffuse proliferative GN, with prominent endocapillary proliferation. The most common infectious agents are *Staphylococcus aureus* and *Streptococcus species*.

This case report may be relevant because the renal dysfunction was highly suggestive of a cryoglobulinemic GN on a clinical ground, but the histologic pattern after performing the renal biopsy oriented towards a different cause of the underlying disease, that required a specific antibiotic treatment. The renal biopsy is always required to confirm a clinical suspicious in patients affected by multiple comorbidities.

## Background

Hepatitis C virus (HCV) may play a pathogenic role in several forms of renal diseases, including immune complex glomerulonephritis (GN) such as cryoglobulinemic membrano-proliferative GN.

This case report may be relevant because the real underlying disease was found to be different from what we thought and it was revealed thanks to the fundamental contribution of the renal biopsy. Its clinical and laboratory features may hide a different renal disease.

## Case presentation

**Fig. 1:**
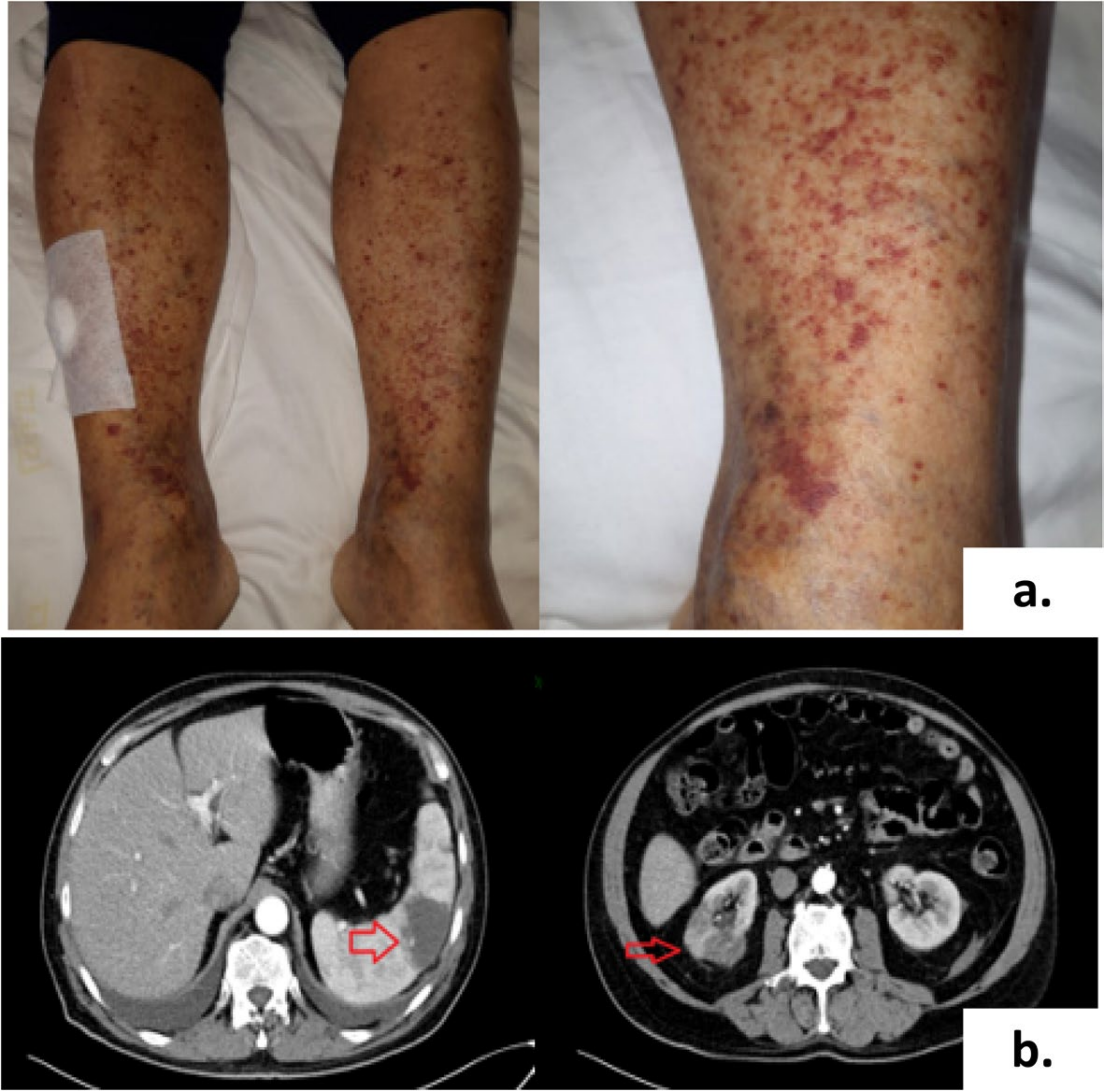
**a**. Purpura-shaped pigmentation in lower limbs bilaterally. **b**. CT-scan of the abdomen and pelvis showing infarction areas presumably due to septic emboli in spleen and right kidney

A 68-year-old man presented at the Department of Nephrology of the Vito Fazzi Hospital in Lecce on May 2019 complaining fever, appetite loss, arthralgias and leg edema that had persisted for a month, accompanied by purpura on the bilateral lower limbs (Fig. 1a). The patient exhibited laboratory tests that showed non-nephrotic proteinuria and progressively decreased renal function in the previous three weeks.

Physical examination on admission showed a body temperature of 38.3 °C, blood pressure 150/90 mmHg, pulse rate 115 beats per minute, respiratory rate 26 breaths per minute and the oxygen saturation 96%. He complained no symptoms or signs of vasculitis on eyes, ears and upper respiratory tract. The laboratory data at the patient’s first presentation are listed in Table 1, indicating anemia, hypoalbuminemia, renal failure with nephrotic proteinuria, and positive inflammatory lab tests. The patient was HCV positive (genotype 2b). No hepatic impairment was detected through routinely blood tests. Moreover, the patient underwent an ultrasound evaluation that revealed regular hepatic features and spleen size, as well as no signs of portal hypertension. Ultrasound elastosonography was also performed with a result not indicative of hepatic fibrosis. Urinalysis revealed microhematuria and proteinuria.

**Table 1:** Laboratory test at the admission

Parameter	Value	Normal range
White blood cells (× 109/L)	9.1	3.5–9.5
Haemoglobin (g/L)	10.7	115–150
Platelet count (× 109/L)	189	125–350
Creatinine (µmol/L)	172.4	44–106
Proteinuria (g/24 h)	3	< 0.15
Albumin (g/dL)	2.3	40–55
Alanine aminotransferase (ALT) (U/L)	37	10–55
Aspartate aminotransferase (AST) (U/L)	28	10–40
Gamma glutamyl transferase (GGT) (U/L)	55	5–40
Prothrombin time (seconds)	12	11–13.5
HBsAg	negative	0–0.05
Anti HBs	negative	negative
Anti HBc	negative	negative
HCV-RNA (IU/mL)	3.29 × 10^4^	< 1.0e2
Rheumatoid factor (IU/mL)	536.6	< 20
Complement C4 (mg/dL)	2	16–38
Complement C3 (mg/dL)	112	79–152
ANA	< 1:100	< 1:100
IgG (g/L)	1073	7.51–15.6
IgM (g/L)	564	0.46–3.04
IgA (g/L)	107	0.82–4.53
Reactive C protein (mg/dL)	300	5–10
ANCA	negative	negative
Cryoglobuline test	4%	negative

*Ig* immunoglobulin, *HbsAg* hepatitis B surface antigen, *ANA* anti-nuclear antibody, *HCV* hepatits C virus, *ANCA* anti-neutrophil cytoplasmic antibodies

The patient was given intravenously human albumin, high-dose furosemide and ceftriaxone after collecting blood and urine specimens for culture. In the following days, anaemia worsened, and a blood transfusion was required, while the respiratory conditions deteriorated. The quantitative cryoglobulin test turn out positive (cryocrit 4%). A biopsy of the cutaneous purpuric lesion was performed that showed epidermal atrophy with hyperpigmentation of the basal layer and mild superficial perivascular lymphocyte infiltrate. Initial therapy consisted of albumin, high-dose iv furosemide and ceftriaxone after collecting blood and urine specimens for culture.

**Fig. 2:**
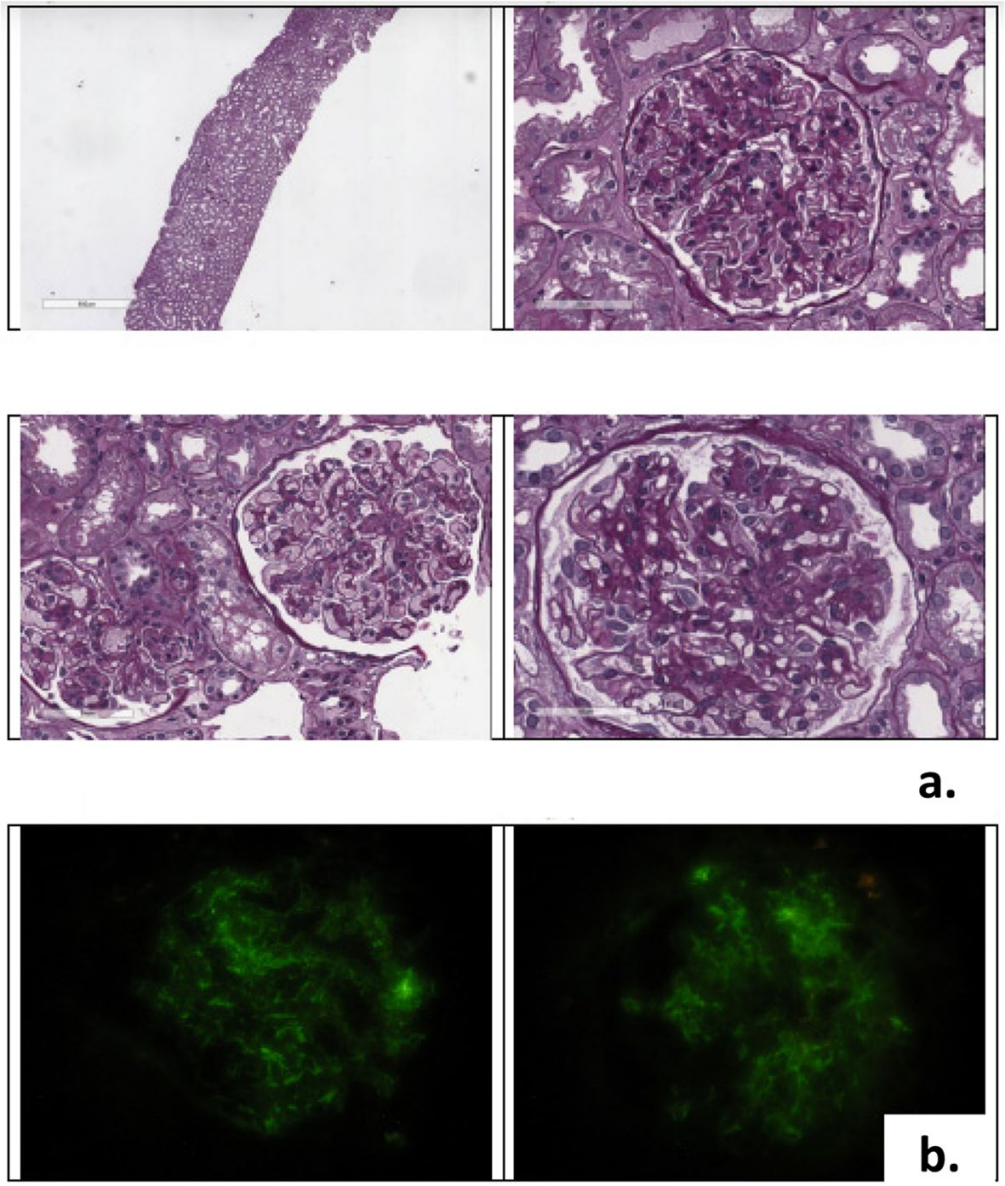
**a**. Renal tissue. Light microscopy shows a diffuse endocapillary proliferative glomerulonephritis. PAS staining. **b**. Renal tissue. Immunofluorescence. Bright mesangial C3 and IgM

A renal biopsy was performed, the histologic light microscopy examination showed mesangial and endocapillary hypercellularity, and expansion of the mesangial matrix. Mild tubular atrophy, interstitial fibrosis and focal inflammatory cell infiltration were also observed (Fig. 2a). There was no thrombosis in capillary lumens. Immunofluorescence revealed granular deposits of IgM and C3 in capillary loops and mesangium (Fig. 2b). The pathological diagnosis was focal endocapillary proliferative GN.

The echocardiography showed aortic valve regurgitation with a 16 × 6 mm unique vegetation. Moreover, blood cultures were found to be positive for penicillin-susceptible Streptococcus viridans, thus the patient suffered from subacute IE. A CT-scan revealed triangular-shaped hypodensity areas in the spleen and in the right kidney, attributable to splenic and kidney infarcts (Fig. 1b).

**Fig. 3:**
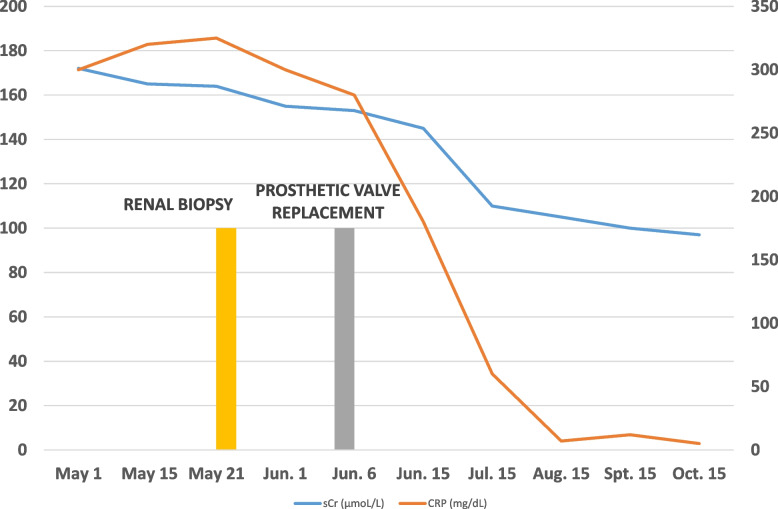
Clinical course during the hospitalization and after the discharge

We immediately started gentamicin in association with ceftriaxone and requested a referral to the Cardiac Surgeon. A biologic prosthetic valve replacement was performed without complications. Successful treatment of infectious endocarditis ameliorated hypocomplementemia and renal failure. An overview of clinical course is provided in Fig. 3.

After an evaluation by the Infectious Diseases Specialist, we decided to wait for the improvement of the renal function before starting the HCV eradication treatment. Therefore, it was performed ten months after the hospital discharge with glecaprevir/pibrentasvir.

At the last follow up visit, the patient was asymptomatic and in apparent healthy status and the laboratory data indicated: serum creatinine of 106 µmol/L, proteinuria of 0.109 mg/24 h, C4 of 32 mg/dL.

## Discussion and conclusions

The past history and clinical data at the time of admission of the presented case were highly suggestive of cryoglobulinemic GN. Mixed cryoglobulinemia-associated GN was usually the most important extrahepatic manifestation of chronic HCV infection before the dramatic modification of the natural history of the disease determined by the new direct acting antiviral drugs. Major clinical diagnostic criteria were: i. serum cryoglobulin; ii. hematuria, proteinuria and renal damage; iii. renal histology showing often a membranoproliferative pattern [1]. Our patient at the time of admission had not been treated with specific anti-HCV therapy; he showed the first two points, together with the clinical suspicion exerted by cutaneous purpura, high levels of HCV-RNA and reduction of serum C4.

Renal biopsy findings ruled out that diagnosis. The histopathologic features at the light microscopy and the immunofluorescence showed IgM and C3 deposits, while IgG deposition was absent. These features were atypical for a kidney involvement caused by cryoglobulins [1]. Instead, GN associated with systemic infections such as IE, especially if secondary to staphylococcus or streptococcus infection, may present a histopathological picture as in the present case [2].

GN secondary to bacterial infection has long been recognized as a form of kidney damage [3, 4]. More specifically, renal involvement during IE may occur with various renal lesions, including renal infarction due to septic emboli and acute or subacute postinfectious GN [2–5].

Our patient did not present a cryoglobulinemic membrano-proliferative GN as we thought initially, since all the initial data were suspicious of an HCV-related renal involvement. Instead, the histological findings demonstrated an IE-associated kidney damage [6, 7].

Sethi suggested that an infectious trigger may promote a Complement alternative pathway-mediated disease process. A similar pattern may be classified as post-infectious GN [8]. Even if C3 GN often shows a membrano-proliferative pattern, some of them may be masqueraded as acute infectious GN [9]. Serum levels of C3 in this patient were normal.

Our patient suffered from subacute IE from one of the species predominantly involved (Streptococcus spp.), associated with septic splenic and renal embolism that are often described in this condition [2]. It has been reported that some of GN associated with IE and glomerular involvement linked with visceral infection are often accompanied by dominant C3 deposition similar to that in our case.

Therefore, unlike the major part of GN related to streptococcal infection, the renal damage in our patient was seen concomitantly with an active infectious process, and thus treatment was based on antibiotic therapy. The patient reached a complete clinical remission of the disease, in contrast to the observed remission rate in adult infection-associated GN that is 26–56% [10–12].

This case emphasizes the broad differential diagnosis of renal involvement in patients affected by multiple comorbidities. Atypical clinical presentation often may issue complex diagnostic challenges and highlights the key role of renal biopsy.

## Data Availability

Patient’s data are regularly recorded in a paper medical record. If needed, pages may be copied into a pdf file.

## Abbreviations

HCV: hepatitis C infection
GN: glomerulonephritis
IE: infectious endocarditis
